# Drivers of carbon flux in drip irrigation maize fields in northwest China

**DOI:** 10.1186/s13021-021-00176-5

**Published:** 2021-04-30

**Authors:** Hui Guo, Sien Li, Fuk-Ling Wong, Shujing Qin, Yahui Wang, Danni Yang, Hon-Ming Lam

**Affiliations:** 1 Center for Agricultural Water Research in China, China Agricultural University, Beijing, 100083 China; 2 Center of Soybean Research of the State Key Laboratory of Agrobiotechnology and School of Life Sciences, The Chinese University of Hong Kong, Hong Kong, China

**Keywords:** Carbon flux, Drip-irrigated maize field, Gross primary productivity, Net ecosystem productivity, Ecosystem respiration, Net biome productivity

## Abstract

**Background:**

Under the escalating threat to sustainable development from the global increase in carbon dioxide concentrations, the variations in carbon flux in the farmland ecosystem and their influencing factors have attracted global attention. Over the past few decades, with the development of eddy covariance technology, the carbon fluxes of farmlands have been determined in many countries. However, studies are very limited for drip irrigation maize the arid regions in northwestern China, which covers a large area where a mixed mode of agriculture and grazing is practiced.

**Results:**

To study the effects of drip irrigation on the net ecosystem productivity (NEE), ecosystem respiration (ER), gross primary production (GPP) and net biome productivity (NBP) in the arid regions of northwestern China, we measured the carbon flux annually from 2014 to 2018 using an eddy covariance system. Our results showed that the maize field carbon flux exhibited single-peak seasonal patterns during the growing seasons. During 2014–2018, the NEE, ER and GPP of the drip-irrigated maize field ranged between − 407 ~ − 729 g C m^−2^, 485.46 ~ 975.46 g C m^−2^, and 1068.23 ~ 1705.30 g C m^−2^. In four of the 5 study years, the ER released back to the atmosphere was just over half of the carbon fixed by photosynthesis. The mean daily NEE, ER and GPP were significantly correlated with the net radiation (Rn), air temperature (Ta), leaf area index (LAI) and soil moisture (SWC). The results of path analysis showed that leaf area index is the main driving force of seasonal variation of carbon flux. When harvested removals were considered, the annual NBP was − 234 g C m^−2^, and the drip-irrigated maize field was a carbon source.

**Conclusions:**

This study shows the variation and influencing factors of NEE, ER and GPP in the growth period of spring maize under film drip irrigation in arid areas of northwest China. The ecosystem was a carbon sink before maize harvest, but it was converted into a carbon source considering the carbon emissions after harvest. The variation of carbon flux was influenced by both environmental and vegetation factors, and its leaf area index was the main driver that affects the seasonal variation of carbon flux.

## Background

With the increasing global atmospheric carbon dioxide (CO_2_) concentration, the carbon cycle has become a hot issue in all fields of research. According to FAO statistics, croplands account for approximately 11% of the world's total land area [14]. Eddy covariance system is an observation technique based on the theory of atmospheric turbulence, which can be used to measure the earth-air exchange process. With the development of eddy covariance technology over a long period of time, so far, technicians have developed a high-precision eddy covariance system, which can realize high frequency and long-term observation of water and carbon fluxes. Based on eddy covariance system, studying how to maintain the balance in the carbon budget of terrestrial ecosystem, in particular farmland, has important scientific significance.

The carbon fluxes in farmland ecosystems are directly affected by human activities, such as irrigation methods, planting patterns and agronomic measures, and these activities in turn influence global carbon fluxes due to the relatively high percentage of land areas devoted to farming [20, 30, 38]. Therefore, reducing carbon emissions from farmland ecosystems can have a significant impact on mitigating climate change. In agriculture, to date, reducing CO_2_ emissions has primarily meant prohibiting the burning of straw and other crop wastes as well as changing farming practices [10, 24, 25, 37]. Study have shown that drip irrigation with plastic mulching significantly increased soil CO_2_ emissions [46]. However, drip irrigation can promote the growth of crops and improve the photosynthetic capacity of crops. In recent years, the fluctuation in farmland ecosystem carbon emissions has become a major concern [22, 23, 36]. A research have shown that the application of organic amendments along with inorganic fertilizers improved net ecosystem carbon budget [7]. Beacuse of the long fallow periods of cotton, cotton cropping system functions as a large carbon source [32]. The wheat–maize rotation system showed a carbon sink with rotation [32]. Compared with traditional tillage, reduced tillage could reduce soil respiration rate to some extent [3]. The results of a long-term situ experiment showed that the net ecosystem exchange of maize was negative, but its net biome productipn was positive (NBP remained positive indicating a carbon net loss) [13].

Carbon flux has obvious seasonal variations. The seasonal variations of NEE is closely related to crop growth [27, 35]. Gross primary production (GPP) is the world’s most important mode of carbon flux and is closely related to ER and biomass accumulation [6]. Factor affecting the carbon flux can be divided into biological and non-biological factors, biological factors mainly refers to the associated with plant growth. The study about carbon flux found that the canopy greenness and coverage is closely related to the spatial and temporal variations of ecosystem carbon flux [42], and plant growth period length to a certain extent determines the value of carbon flux in different seasons [12]. The response of carbon flux to environmental factors is different. In the early growth stage of plant in arid regions, precipitation is the main factor affecting net ecosystem exchange [34].

In recent years, drip irrigation has been actively promoted in the arid areas of northwestern China as a water-saving agricultural technology. Drip irrigation under film can provide a timely and appropriate amount of fertilizer and irrigation according to different needs, and it is one of the important measures used to couple water and fertilizer. According to the published statistics, drip irrigation can save 40–60% water and 30–50% fertilizer [31]. At present, using drip irrigation technology under film has been popularized on more than four million hectares in China, and it has been applied to the cultivation of more than 40 crops, among which wheat, maize, cotton and other major field crops have an average yield increase of more than 30% [18]. Drip irrigation under film is expected to replace the traditional method of border irrigation. Gansu Province is the largest seed production base for hybrid maize in China. At present, there are few studies on the changes in NEE, ER and GPP in the farmland ecosystem under drip irrigation. Research on the global carbon balance must cover all types of biomes, including maize-production areas in the arid regions of northwestern China.

The purpose of the study is to quantify the NEE, GPP and ER of the maize crop ecosystem in the arid regions of northwestern China using eddy covariance systems. The specific goals are as follows: (1) quantify the seasonal and interannual variations in carbon flux in this region, (2) identify the primary environmental factors affecting the seasonal variations of carbon flux, and (3) quantify the growing season carbon budget of drip-irrigated spring maize fields.

## Materials and methods

### Site description

**Fig. 1:**
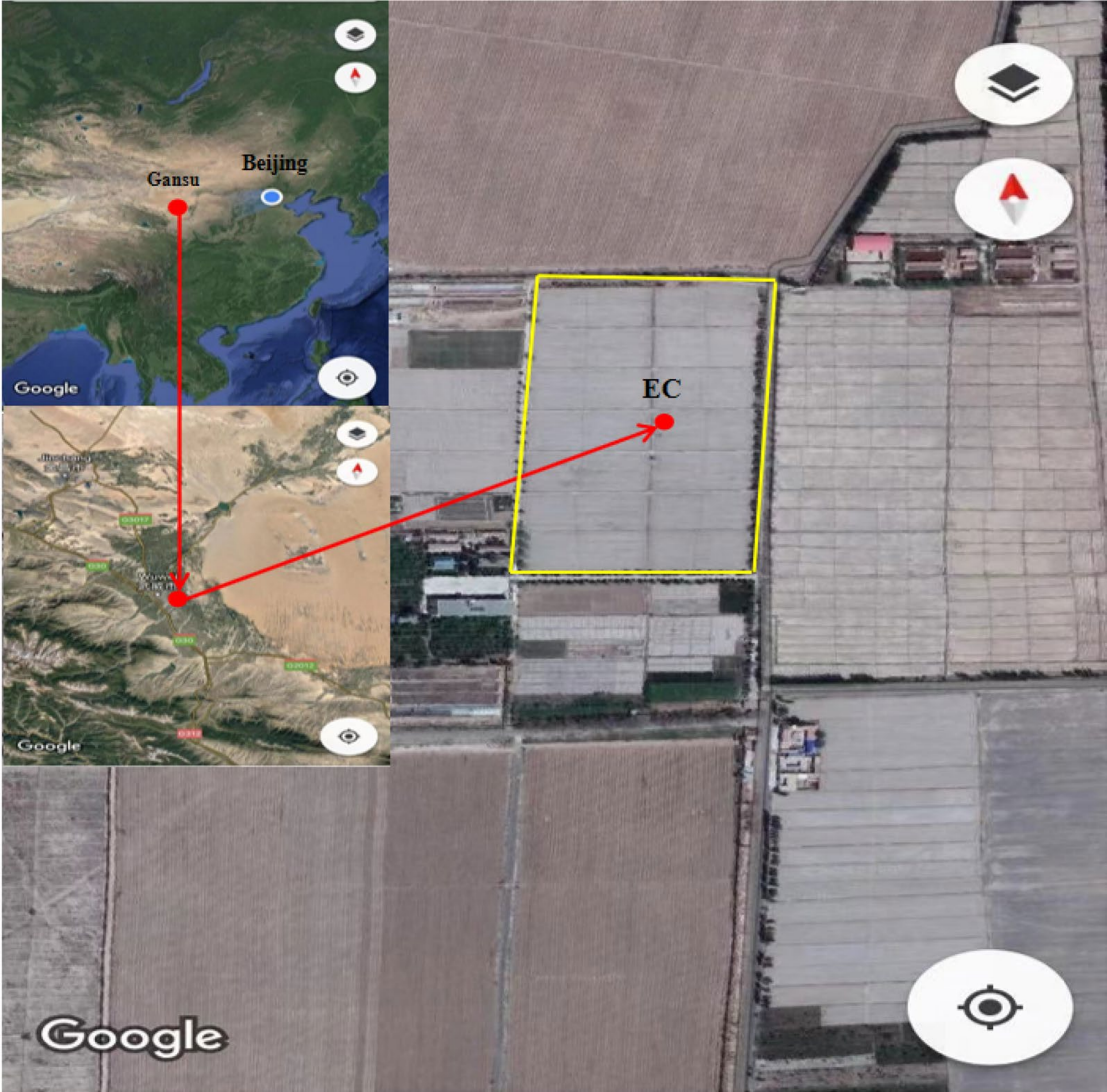
Location diagram of the experimental area [19]

This study was conducted east of Hexi Corridor in the arid area of Northwest China (37°52′ N, 102°50′ E, 1585 m elevation) at the border of Tengger Desert. This area has a typical continental climate and strong temperature differences among the four seasons. The annual average temperature is 7.8 °C. Water resources are scarce in this region; the annual total precipitation is 160 mm, the annual evaporation is more than 2000 mm, and the groundwater depth is 40–50 m. The soil in the test area is sandy loam [28]. The soil texture at 0–0.8 m deep is silty loam [28, 29], the average soil dry bulk is 1.52 g cm^−3^, and the average field capacity is 0.29 m^3^ m^−3^. Before maize sowing, the PH of 0–30 cm in the experimental area was 8.1, and the content of soil organic matter was 7.6 g kg^−1^. The entire experimental area has been cultivated for many years, its length is 400 m and its width is 200 m. Refer to Fig. 1 for detailed locations. The crop cultivated here for seed production is spring maize, and it is sown in late April and harvested in mid-September. The irrigation method is drip irrigation under film mulch. The irrigation and fertilization conditions from 2014 to 2018 are shown in Table 1 and Table 2.

**Table 1:** The details of irrigation in maize field from 2014 to 2018

Date	Irrigation (mm)	Date	Irrigation (mm)	Date	Irrigation (mm)	Date	Irrigation (mm)	Date	Irrigation (mm)
2014/6/6	50.00	2015/6/1	50.00	2016/4/24	51.00	2017/4/23	35.10	2018/5/1	50.77
2014/6/23	50.00	2015/6/13	50.00	2016/6/10	45.47	2017/6/14	68.02	2018/6/7	53.08
2014/6/28	50.00	2015/6/26	50.00	2016/6/25	49.82	2017/6/25	64.79	2018/6/18	40.58
2014/7/7	50.00	2015/7/10	50.00	2016/7/3	54.26	2017/7/10	62.07	2018/6/30	49.62
2014/7/18	50.00	2015/7/24	50.00	2016/7/15	65.09	2017/7/22	57.45	2018/7/11	55.38
2014/7/28	50.00	2015/8/7	50.00	2016/7/27	54.45	2017/8/8	53.76	2018/7/22	50.96
2014/8/22	50.00	2015/8/20	50.00	2016/8/7	53.53	2017/8/22	27.09	2018/8/1	68.27
		2015/8/31	50.00	2016/8/22	53.19			2018/8/14	53.27

**Table 2:** The details of fertilization in maize field from 2014 to 2018

Year	Date	(NH4)_2_HPO_4_ (kg hm^−1^)	Urea (kg hm^−1^)	Compound fertilizer (kg hm^−1^)
2014	10-Apr (base fertilizer)	275.00	42.00	
	8-Jun		150.00	
	5-Jul		120.00	
2015	12-Apr (base fertilizer)	262.50	37.50	
	30-May	63.00	9.00	
	11-Jun	78.75	11.25	
	25-Jun	168.00	24.00	
	9-Jul	126.00	18.00	
	27-Jul	105.00	15.00	
2016	5-Apr (base fertilizer)	300.00	225.00	
	27-Jun		300.00	
	15-Jul		150.00	
2017	26-Mar (base fertilizer)	375.00		375.00
	15-Jun		270.00	
	30-Jun		180.00	
2018	26-Mar (base fertilizer)	375.00		375.00
	7-Jun		225.00	
	18-Jun		150.00	
	30-Jun		75.00	

### Flux and climatic-factor measurements

As a common technique for monitoring water vapor flux and carbon dioxide flux between the surface and the atmosphere, eddy covariance technology has become increasingly refined after decades of theoretical development and practical application [4]. An open-path eddy covariance system was installed in the center of a homogenously vegetated area covering approximately 8 ha. The eddy covariance system consisted of an open-path infrared gas analyzer (EC150, Campbell Scientific Inc., USA) and a three-dimensional anemometer (CSAT3, Campbell Scientific Inc., USA), both installed at a height of 3 m above the ground.

Routine meteorological factors were measured simultaneously. Air temperature, relative humidity and saturated vapor pressure deficit were measured by a temperature and humidity probe at 3 m (HMP155A, CSI, USA). Radiation was monitored by a radiation meter (CNR4, Kipp & Zonen, Holland). Soil temperature probes (109L, Campbell Scientific Inc., USA) and soil moisture probes (CS616, Campbell Scientific Inc., USA) were installed to monitor the variations in the soil temperature and soil water content (SWC), respectively, at depths of 20 cm, 40 cm, 60 cm, 80 cm and 100 cm. All the data were collected with a CR3000 (Campbell Scientific Inc., USA) data logger. Precipitation (P) was obtained from a standard weather station (HOBO, Onset Computer Corp, USA) installed in the experimental station, and irrigation amount was measured by water meter.

To compensate for the heterogeneity of the underlying vegetation and the error due to instrument installation variability, the raw data were normalized to improve the comparability of the final results. We used Loggernet (Campbell Scientific Inc., USA) to convert the collected data with a sampling frequency of 10 Hz into data with a frequency of 30 min. Eddy Pro software (Li-COR, USA) was then used to perform the stability test, atmospheric turbulence heat verification, and other analyses. The stability of the nighttime atmospheric conditions lowered the data quality. In the case of weak turbulence, the results obtained using the eddy covariance method do not accurately reflect the real carbon exchange of the underlying surface. The criterion that reflects the strength of the turbulence in the atmosphere is the frictional wind speed. According to the average values test method [45], for each year from 2014 to 2018, the critical frictional wind speed values were 0.15 m s^−1^, 0.15 m s^−1^, 0.20 m s^−1^, 0.18 m s^−1^ and 0.12 m s^−1^, respectively. Therefore, when the data were processed, the nighttime carbon flux data associated with a corresponding frictional wind speed that was less than the critical frictional wind speed were removed. The data outliers were removed since they were often caused by external factors such as rain and snow or unstable voltage. In field operations, the installation of the instrument cannot be guaranteed to be absolutely perpendicular to the ground, so the data must be tilted for correction, that is, there must be a coordinate rotation. Finally, a frequency loss correction and an air density correction are required.

Due to instrument failure, extreme weather, power supply issues and data processing errors, there were missing data. To achieve data continuity and integrity, the data were interpolated. Data gaps due to turbulent fluxes or instrument malfunction were divided into short gaps (< 2 h) and long gaps (> 2 h) [15]. The former were filled by linear interpolation, and the latter were filled using statistical and empirical models [5]. The Michaelis–Menten equation was used for daytime data gaps [33]:

1 $$NEE = ER - \frac{{\alpha \cdot PAR \cdot P_{\max } }}{{P_{\max } + \alpha \cdot P_{\max } }}$$

where *ER* is the dark respiration, *PAR* is the photosynthetically active radiation, *α* (umol CO_2_ μmol PAR^−1^) is the apparent quantum efficiency, and *P*_*max*_ (umol CO_2_ m^−2^ s^−1^) is the maximum ecosystem photosynthesis rate.

The vant Hoff equation was used for nighttime data gaps [11]:

2 $$ER = ER_{ref} \exp \left( {B(T_{s} - T_{ref} )} \right)$$

where *ER*_*ref*_ is the reference ER at 10 °C, *B* is the regression parameter, *T*_*s*_ is the surface temperature and *T*_*ref*_ is the reference surface temperature at 10 °C.

### Calculation of the leaf area index (LAI)

The leaf surface area was measured every seven to 10 days from the seedling stage to crop harvest. In the field, we chose six different locations with nine representative plants at each site. A measuring tape (Minimum scale: mm) was used to measure the length and width of each leaf. The LAI of the maize leaves can then be obtained using the following formula (Eq. 1) [19]:

3 $$LAI = 0.74 \times \frac{{\sum\limits_{i = 1}^{n} {L_{i \times } W_{i} } }}{D \times S}$$

where *LAI* is the leaf area index of maize, 0.74 is the empirical constant, *Li* is the length of leaf *i*, *Wi* is the width of leaf *i*, and *D* and *S* are the distance between two rows and the space between two plants, respectively.

### Flux partitioning

NEE is the net ecosystem exchange (it has a negative value in this context, representing net CO_2_ fixation by the ecosystem). ER includes both autotrophic and heterotrophic respiration [9]. Autotrophic respiration includes the respiration of both the underground and aerial parts of the maize plant, and heterotrophic respiration refers to the respiration of the soil organisms. GPP represents the amount of CO_2_ assimilated by the maize plants during photosynthesis, the GPP value is equal to the difference between ER and NEE.

Crops do not conduct photosynthesis at night, i.e., GPP = 0. Therefore, the NEE measured by the eddy covariance system at night is the ER of the farmland ecosystem [8]. Once the relationship between the nighttime NEE and surface temperature (T_s_) was established, the daytime ER was obtained by plugging the daytime T_s_ data into the equation. The most commonly used method is to use the respiratory model to interpolate the missing data. We used the van’t Hoff model to simulate the nighttime CO_2_ flux of the maize with drip irrigation under film mulch as Eq. (2).

The difference between the NEE and the calculated ER is GPP.

4 $$GPP = ER - NEE$$

### Net biome productivity

Net biome productivity (NBP) is defined as:

5 $$NBP = C_{i} - C_{e} - NEE$$

where *C*_*i*_ is imported carbon, *C*_*e*_ is exported carbon. When the value of NBP is positive, it means the ecosystem is a carbon sink. Otherwise, it is the carbon source.

The amount of exported carbon can be calculated based measured data for biomass, as follows:

6 $$C_{e} = D_{g} *a_{1} + D_{c} *a_{2} + D_{l} *a_{3} + D_{s} *a_{4}$$

where *D*_*g*_ is the dry grain, *D*_*c*_ the dry cob, *D*_*l*_ the dry leaf and *D*_*s*_ the dry stem, *a*_*1*_, *a*_*2*_* a*_*3*_ and *a*_*4*_ is the carbon percentage of different organs, the values of *a*_*1*_, *a*_*2*_* a*_*3*_ and *a*_*4*_ were 0.447, 0.468, 0.452 and 0.452, respectively [21, 44]. In our experimental area, the grain and cob were harvested completely, the roots was left in the field, and about 10% of the leaves and stems were left in the field. The values of *D*_*g*_, *D*_*c*_*, D*_*l*_, *D*_*s*_ and *C*_*e*_ are shown in Table 3.

**Table 3:** Dry grain (D_g_), dry cob (D_c_), dry leaf (D_l_), dry stem (D_s_) and exported carbon (C_e_) in the spring maize field during 2014–2018

Year	D_g_ (g m^−2^)	D_c_ (g m^−2^)	D_l_ (g m^−2^)	D_s_ (g m^−2^)	C_e_ (g C m^−2^)
2014	904	126.43	314.70	502.63	795.75
2015	997	139.44	310.42	724.63	931.98
2016	1095	153.15	432.91	596.09	979.73
2017	529	73.99	192.40	614.35	599.27
2018	792	110.77	372.28	492.23	757.55
AVG	863.40	120.76	324.54	585.99	812.86

### Statistical analysis

All the statistical analyses were performed using SPSS for Windows Software (Version 18.0, SPSS Inc., Chicago, IL, USA). Simple linear regression was used to evaluate the relationships between daily NEE, ER and GPP. Multiple regression analysis was used to analyze the relationships between various environmental factors ans leaf area index and NEE, ER and GPP.

## Results

### Seasonal variations in meteorological and vegetation factors

**Fig. 2:**
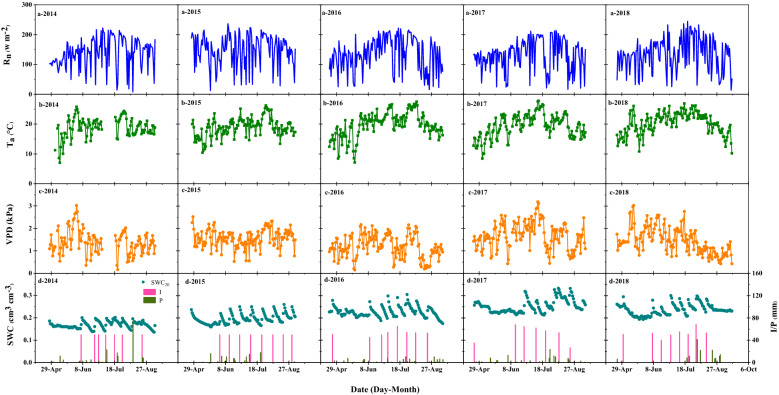
Mean daily values of meteorological factors measured at the experimental sites in growing seasons during 2014–2018: **a** net radiation (R_n_), **b** air temperature (T_a_), **c** saturated vapor pressure deficit (VPD), **d** soil water content (SWC) and precipitation and irrigation (P + I)

Figure 2 shows the seasonal variation characteristics of net radiation (Rn), air temperature (Ta) saturated water vapor pressure difference (VPD), soil water content at 0–20 cm (SWC) and precipitation (P) and irrigation (I) in the study site from 2014 to 2018. The seasonal fluctuations in Rn were small. Rn remained at a high level from May to August but began to decline slowly after September (Fig. 2a). The average Rn over the entire growing period were 141.14, 150.26, 136.51, 132.28 and 133.27 w m^−2^ in 2014–2018, respectively. Among then, the average Rn values peaked at the heading stage or shooting stage. The average air temperatures for each of the five growing seasons were 19.02, 19.12, 21.01, 19.58 and 20.14 °C, respectively (Fig. 2b). During maize growth and development, the pattern of temperature during each growth period differed slightly among years. In 2014, 2017 and 2018, the air temperature peaked during the heading stage, while in 2015 and 2016, it peaked during the filling stage. The difference in the VPD between the years was rather small. However, in 2017, the seasonal average VPD was the highest because that year had the lowest amount of precipitation and irrigation, which led to dry air. The soil water content for the five growing seasons were consistent with precipitation and irrigation events, and the values were 0.17, 0.20, 0.23, 0.25 and 0.24 cm^3^ cm^−3^, respectively. The sum of precipitation and irrigation were 545.40 mm, 519.40 mm, 542.22 mm, 502.28 mm and 578.32 mm, respectively. Since this is an arid region, precipitation played a very minor role in supplying the water required for crop growth in comparison to irrigation.

**Fig. 3:**
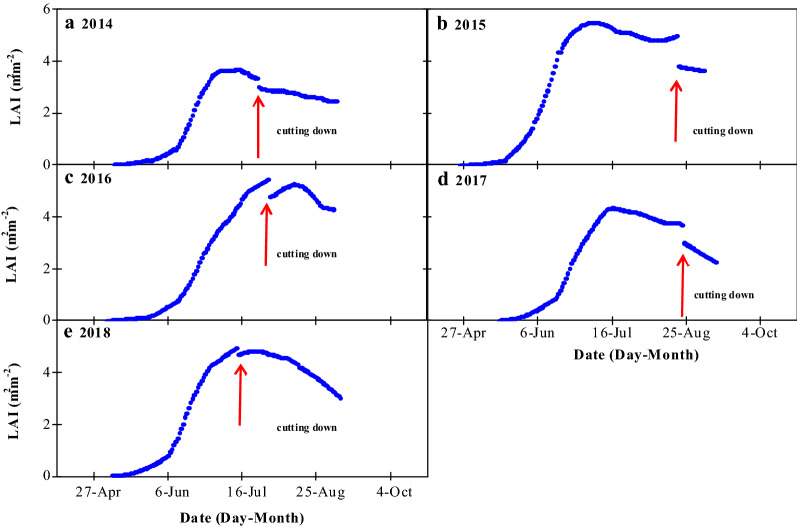
Variations in the leaf area index (LAI) of maize during the growing seasons, 2014–2018

Figure 3 shows the seasonal variation patterns in the maize LAI from 2014 to 2018. Throughout the growing season, the change in LAI assumed a parabolic curve. Starting from the seedling stage, LAI increased with the crop growth and peaked at the heading stage. During the mid and late stages of maize growth, LAI decreased significantly because of the special management measures that were implemented for seed maize, i.e., after pollination, the male plants were cut, which led to a significant decrease in LAI. For each year from 2014 to 2018, the maximum LAI values were 3.09, 5.53, 5.28, 4.26 and 4.93 m^2^ m^−2^, respectively. The maximum LAI for 2015 was the highest among all 5 years, which was primarily due to the best crop growth, which was observed in 2015.

### Seasonal and interannual variations of NEE, ER and GPP

**Fig. 4:**
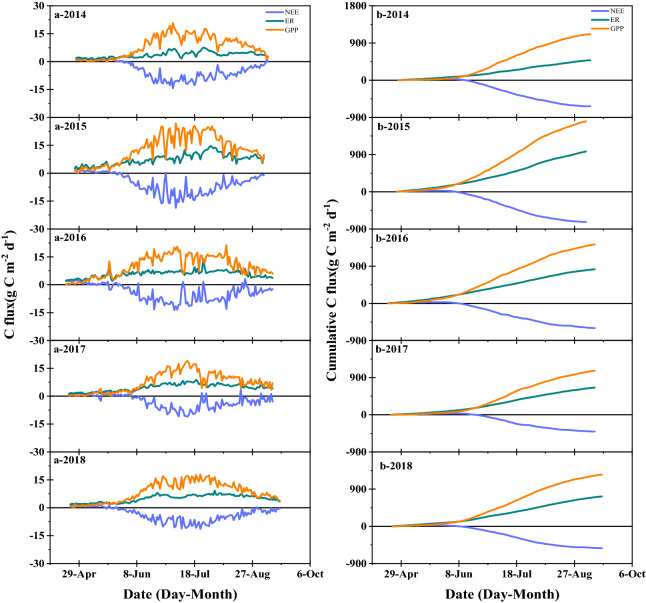
Seasonal fluctuations in daily average **a** net ecosystem exchange (NEE), ecosystem respiration (ER) and gross primary productivity (GPP), **b** the cumulative NEE, ER and GPP over the period of 2014–2018

The seasonal dynamics of NEE, GPP and ER during 2014–2018 are illustrated in Fig. 4. With the growth of the maize and variations of climate, NEE, ER and GPP showed clear seasonality. The variations in ER were relatively weak, whereas large fluctuations in GPP during crop growth were frequent. In the early growth stage, ER was low, mainly due to the low air temperature. Daily ER increased significantly in mid-June due to both the increase in air temperature and crop growth. The maximum value of ER occurred during the heading or filling stage; with crop decline and the reduction of temperature, ER began to slowly decline. Daily GPP and NEE peaked in the heading stage in all 5 years; at this stage, leaf area reached its maximum value, and the meteorological conditions were optimal for growth.

During the early stage of crop growth, i.e., from April to May, the NEE was positive, indicating that the total respiration per sq. m. for the maize field was higher than the total photosynthesis. Thus, the maize field released CO_2_ into the atmosphere. During the fast-growth stage, i.e., from June to August, the total photosynthesis exceeded the respiration, and the NEE peaked over a range from − 10 to − 20 g C m^−2^ d^−1^ between 2014 and 2018. Thus, at this stage of growth, maize field was most capable of sequesturing CO_2_ from the atmosphere. During the late stage of crop growth, i.e., from September to October, the NEE value gradually decreased but remained negative, indicating that the carbon absorption capacity of the maize field was weak during this time (Fig. 4). The findings demonstrate that during the early growth stage, the maize field released CO_2_ into the atmosphere. Its CO_2_ absorption capacity gradually increased as the growing season progressed, reaching a peak and then slowly declining at the end of the growing season. From 2014 to 2018, the GPP and NEE values decreased significantly on rainy or cloudy days when the photosynthetic intensity of the crop decreased significantly, thus resulting in a significant reduction in the GPP.

NEE, ER and GPP were not subjected to large inter-annual variability (Table 4). On the annual scale, comparing to NEE and GPP, the cumulative ER in 5 years showed a more significant interannual variability, and the value of CV was 0.22. The cumulative NEE, ER and GPP for the 5 years ranged from to − 406.76 to − 729.89 g C m^−2^, 661.84 to 975.46 g C m^−2^ and 1705.30 to 1068.63 g C m^−2^, respectively (Table 4). The maximum value of NEE, ER, and GPP occurred in the bloom period of crop growth, and the coefficient of variation was similar to the annual, with CV values of 20%, 24%, and 14%, respectively.

**Table 4:** Growing season sums of net ecosystem exchange (NEE), ecosystem respiration (ER) and gross primary productivity (GPP), NEE_max_ is the peak of NEE in the growing season, ER_max_ is the maximum value of ecosystem respiration in the growing season, GPP_max_ is the maximum value of GPP in the growing seasons

Year	Growing days	NEE	ER	GPP	NEE_max_	ER_max_	GPP_max_
	(Day)	(g C m^−2^)	(g C m^−2^)	(g C m^−2^)	(g C m^−2^)	(g C m^−2^)	(g C m^−2^)
2014	134	− 630.13	485.46	1115.54	− 14.32	7.6	20.83
2015	132	− 729.89	975.46	1705.3	− 18.69	14.67	26.79
2016	144	− 601.18	825.81	1427.05	− 13.67	9.98	21.31
2017	142	− 406.76	661.84	1068.63	− 10.98	8.77	18.99
2018	146	− 527.47	724.94	1252.35	− 11.42	9.14	17.94
Mean	139.6	− 579.09	734.7	1313.77	− 13.82	10.03	21.17
SD	5.57	107.89	163.58	232.07	2.75	2.44	3.06
CV	0.04	− 0.19	0.22	0.18	− 0.20	0.24	0.14

*SD* standard deviation, *CV* coefficient of variation

**Fig. 5:**
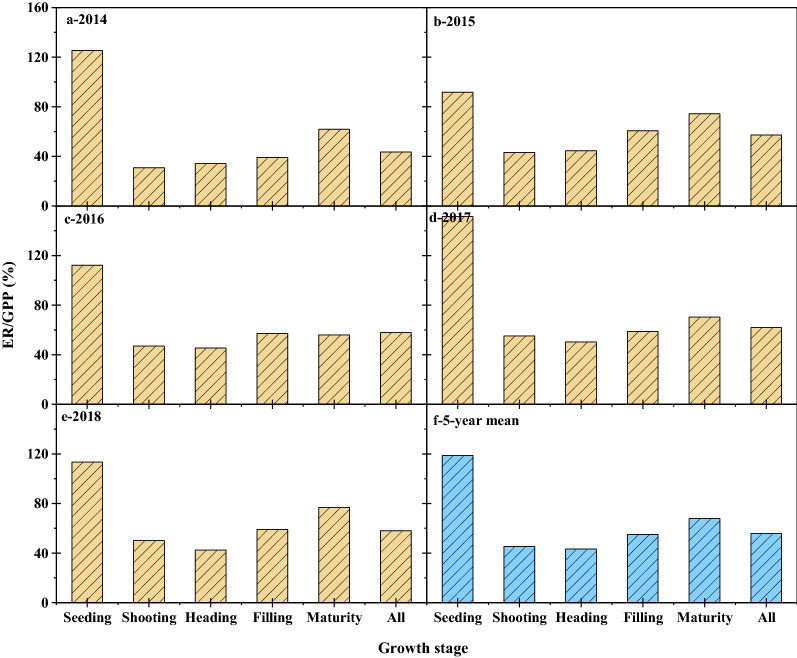
The variation of the ratio of ER/GPP in different growing stages during 2014–2018

The ratio of the ER to the GPP ranged from 44 to 62% in growing seasons of the 5 years, indicating that over half of the carbon fixed via photosynthesis was released back to the atmosphere by respiration. In the early stage of crop growth, the photosynthetic capacity of maize was relatively weak, and the field was mainly dominated by soil respiration. Therefore, the ratio of ER/GPP was relatively high at the seedling stage, exceeding 100% in four of 5 years. As crops growing and development, the ability to photosynthesize increased, and the ratio begins to decline slowly. However, at the later stage of growth, the aging and falling of leaves caused by crop ripening increased the ER/GPP ratio. The year 2014 was an exception in that the ER/GPP ratio was below 50%. During the seedling stage, the ER/GPP ratio exceeded 100% in all years except 2015, which indicated that the amount of CO_2_ released into the atmosphere by ER was greater than the amount fixed by photosynthesis. The 5-year mean values of ER/GPP at different fertility stages were 119%, 45%, 43%, 55% and 68%, respectively (Fig. 5).

**Fig. 6:**
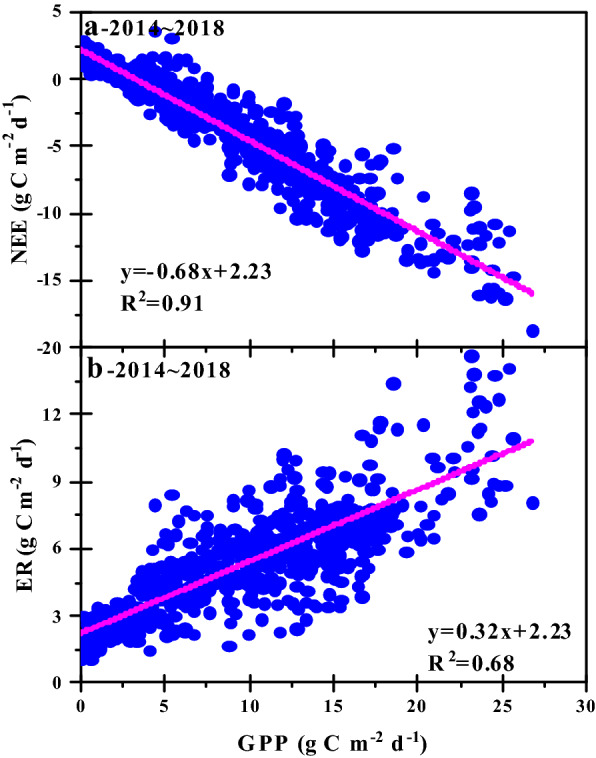
The seasonal variation in ecosystem respiration (ER) and net ecosystem exchange (NEE) in response to gross primary productivity (GPP) during 2014–2018. *Represents a significant level (p < 0.001)

We used a linear regression model to explore the relationship between NEE, ER and GPP. Figure 6 showed that the variation of NEE and ER were significantly correlated with GPP, and both R2 were higher (p < 0.0001). The value of NEE decreased with the increase of GPP, which indicated that the carbon sequestration capacity of crops increased with the increase of photosynthetic capacity. ER increased with the increase of GPP, which also indicated that when the photosynthetic capacity of crops increased, the respiration capacity of maize fields also increased. According to the regression equation (Fig. 6), the variation of GPP contributed 68% and 32% to the variation of NEE and ER, respectively.

### Relationships between environmental variables and NEE, ER and GPP

Analyses of the relationships between each of NEE, ER and GPP and various environmental factors and plant physiology are key to interpreting the seasonal and interannual variations in NEE, ER and GPP in maize fields. The results from the statistical analyses of daily average NEE, ER, GPP, Rn, Ta, VPD, SWC, IP and LAI from 2014 to 2018 are shown in Table 5.

**Table 5:** Partial correlation coefficients between environmental variables and net ecosystem exchange (NEE), ecosystem respiration (ER) and gross primary productivity (GPP)

Carbon flux	Year	Rn	Ta	VPD	SWC	IP	LAI
NEE	2014	− 0.47^a^	− 0.320^a^	0.153	− 0.303^a^	− 0.176^b^	− 0.817^a^
	2015	− 0.390^a^	− 0.470^a^	0.044	− 0.148	− 0.146	− 0.806^a^
	2016	− 0.625^a^	− 0.666^a^	− 0.407^a^	− 0.266^a^	− 0.175^b^	− 0.497^a^
	2017	− 0.581^a^	− 0.638^a^	− 0.449^a^	− 0.148	− 0.109	− 0.749^a^
	2018	− 0.480^a^	− 0.666^a^	− 0.11	− 0.293^a^	− 0.188^b^	− 0.765^a^
ER	2014	0.448^a^	0.395^a^	− 0.037	0.231^a^	− 0.013	0.775^a^
	2015	0.258^a^	0.733^a^	0.350^a^	0.339^a^	0.034	0.742^a^
	2016	0.313^a^	0.675^a^	0.217^a^	0.111	0.05	0.471^a^
	2017	0.329^a^	0.770^a^	0.432^a^	0.272^a^	0.114	0.878^a^
	2018	0.338^a^	0.618^a^	− 0.202^b^	0.384^a^	0.154	0.929^a^
GPP	2014	0.499^a^	0.365^a^	− 0.132	0.303^a^	0.137	0.859^a^
	2015	0.376^a^	0.619^a^	0.106	0.237^a^	0.117	0.858^a^
	2016	0.596^a^	0.748^a^	0.393^a^	0.247^a^	0.154	0.547^a^
	2017	0.523^a^	0.727^a^	0.470^a^	0.204^b^	0.118	0.845^a^
	2018	0.455^a^	0.687^a^	0.001	0.344^a^	0.186^b^	0.892^a^

*ER* ecosystem respiration, *NEE* net ecosystem exchange, *GPP* gross primary productivity, *Rn* net radiation, *Ta* air temperature, *VPD* *vapor* pressure deficit, *SWC* soil water content, *IP* the sum of precipitation and irrigation, *LAI* leaf area index

^a^Significantly correlated at the 0.01 level (bilateral) and ^b^ significantly correlated at the 0.05 level (bilateral)

The results of the 5-year study showed that NEE was correlated with R_n_, T_a_ and LAI. As R_n_, T_a_ and LAI increased and decreased, the smaller the negative value of NEE was, the stronger the ability to fix CO_2_ in the atmosphere was. VPD in 2016 and 2017 also showed a very significant correlation with NEE (p < 0.01), and SWC also had a significant impact on NEE in 2014, 2016 and 2018. However, the effect of irrigation and precipitation on NEE was not significant. The research results on ER showed that during the growing season of maize from 2014 to 2018, ER was significantly positively correlated with Rn, Ta and LAI (p < 0.01). The seasonal variations of ER in 2015–2018 were also influenced by VPD (p < 0.01). In addition to 2016, the influence of SWC on ER is also not negligible. Therefore, Rn, Ta and LAI have important effects on ecosystem respiration. Examination of the relationships between ER and environment/physiological factors showed high correlation coefficients between ER and Ta and ER and LAI (Table 5), indicating that the ER of the farmland ecosystem is sensitive to crop growth and Ta. With crop growth and development, the ER capacity also increased.

During the period of crop growth, LAI was the most important factor affecting GPP, followed by T_a_ and R_n_, which showed a significant positive correlation (p < 0.01). The coefficient of correlation between the GPP and LAI was the highest, indicating that a higher LAI corresponds to more photosynthetic activity by the crop. In addition, the correlation between the GPP and T_a_ was high, revealing that within a given range, the photosynthetic ability of the plants was affected by air temperatures. The 2016 and 2017 VPD also affected the seasonal variations in the GPP.

NEE, ER and GPP in the spring maize growing season all showed significant correlations with net radiation (Rn), air temperature (Ta) and leaf area index (LAI). In addition, we also found that soil water content has a significant effect on GPP. Among all the investigated potential drivers, we found that the leaf area index was the most important controls.

In order to more accurately predict the seasonal variations of NEE, ER and GPP, according to the results in Table 5, we selected the factors with highly significant correlations (p < 0.01) with NEE, ER and GPP for multi-factor fitting. As can be seen from the results in Table 5, when multi-factor regression was adopted, the seasonal variations of NEE, ER and GPP could be well simulated during the growing seasons, among which GPP has the best simulation effect, with goodness of fit values of 0.72–0.78 for NEE, 0.76–0.90 for ER and 0.70–0.90 for GPP (Table 6).

**Table 6:** Regression results of multi− factor liner model between daily carbon flux (NEE, ER and GPP) and environmental variables and LAI, selected according to significant level p < 0.01

Carbon flux	Year	Multi− factor liner model	R^2^	p
NEE	2014	NEE = − 0.02Rn − 0.05Ta − 24.87SWC − 1.50LAI + 8.03	0.73	< 0.01
	2015	NEE = − 0.04Rn − 0.05Ta − 1.83LAI + 7.29	0.78	< 0.01
	2016	NEE = − 0.03Rn − 0.30Ta − 1.17VPD − 10.19SWC − 0.41LAI + 10.22	0.72	< 0.01
	2017	NEE = − 0.03Rn − 0.07Ta − 0.44VPD − 1.42LAI + 5.07	0.74	< 0.01
	2018	NEE = − 0.01Rn − 0.24Ta + 3.44SWC − 1.22LAI + 5.59	0.76	< 0.01
	Total	NEE = − 1.03LAI − 0.029Rn − 1.67Ta − 0.03IP + 5.72SWC	0.79	< 0.01
ER	2014	ER = 0.01Rn + 0.06Ta + 10.06SWC + 0.54LAI − 1.50	0.79	< 0.01
	2015	ER = 0.004Rn + 0.27Ta + 1.60VPD + 3.36SWC + 0.85LAI − 4.38	0.78	< 0.01
	2016	ER = 0.003Rn + 0.47Ta − 2.09VPD − 0.09LAI − 1.16	0.76	< 0.01
	2017	ER = − 0.002Rn + 0.23Ta + 0.03VPD + 1.15SWC + 0.83LAI − 1.32	0.90	< 0.01
	2018	ER = 0.002Rn + 0.09Ta − 0.74SWC + 0.87LAI + 0.77	0.89	< 0.01
	Total	ER = 0.52LAI + 0.17Ta + 0.01Rn + 5.04SWC	0.71	< 0.01
GPP	2014	GPP = − 0.01Rn + 0.04Ta + 17.39SWC + 2.69LAI − 3.36	0.85	< 0.01
	2015	GPP = 0.05Rn + 0.53Ta − 34.61SWC + 2.70LAI − 6.04	0.90	< 0.01
	2016	GPP = 0.04Rn + 0.77Ta − 1.91VPD + 8.70SWC + 0.33LAI − 11.09	0.70	< 0.01
	2017	GPP = 0.02Rn + 0.29Ta + 0.45VPD + 2.26LAI − 6.10	0.86	< 0.01
	2018	GPP = 0.02Rn + 0.33Ta − 4.17SWC + 2.09LAI − 4.82	0.89	< 0.01
	Total	GPP = 1.54LAI + 0.03Rn + 0.34Ta + 0.02IP	0.89	< 0.01

### Carbon budget

**Fig. 7:**
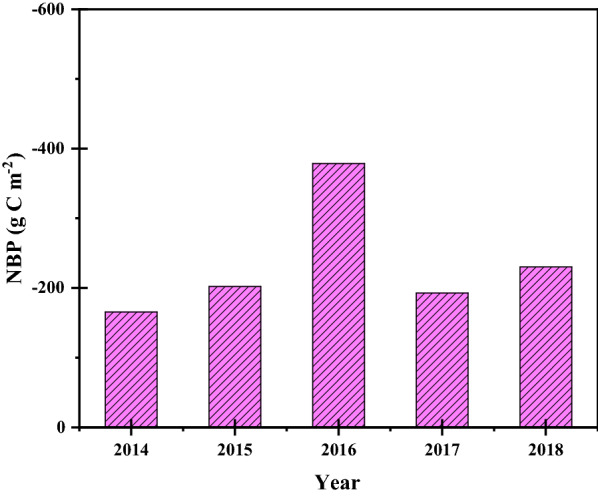
The variation of net biome productivity (NBP) in growing seasons during 2014–2018

Net biome productivity was analyzed to determine whether drip-irrigated maize fields were carbon sources or carbon sinks. According to Eq. 5, it can be known that NBP is the difference between the carbon input and the carbon output and the net ecosystem exchange. Since no organic fertilizer was applied in our experimental area, the total input carbon was 0. From 2014 to 2018, the NBP of the maize field in the experiment site was − 165.12, − 202.09, − 378.55, − 192.51 and − 230.08 g C m^−2^, respectively. In 2016, because the water, fertilizer, light and heat conditions were more suitable for the growing of maize, the maximum yield of maize was 1095 g C m^−2^, so the output of carbon was also the largest. During the drip-irrigated maize field was a carbon source and the average NBP was − 233. 77 g C m^−2^ (Fig. 7).

## Discussion

### Factors controlling seasonal variations in carbon flux

**Fig. 8:**
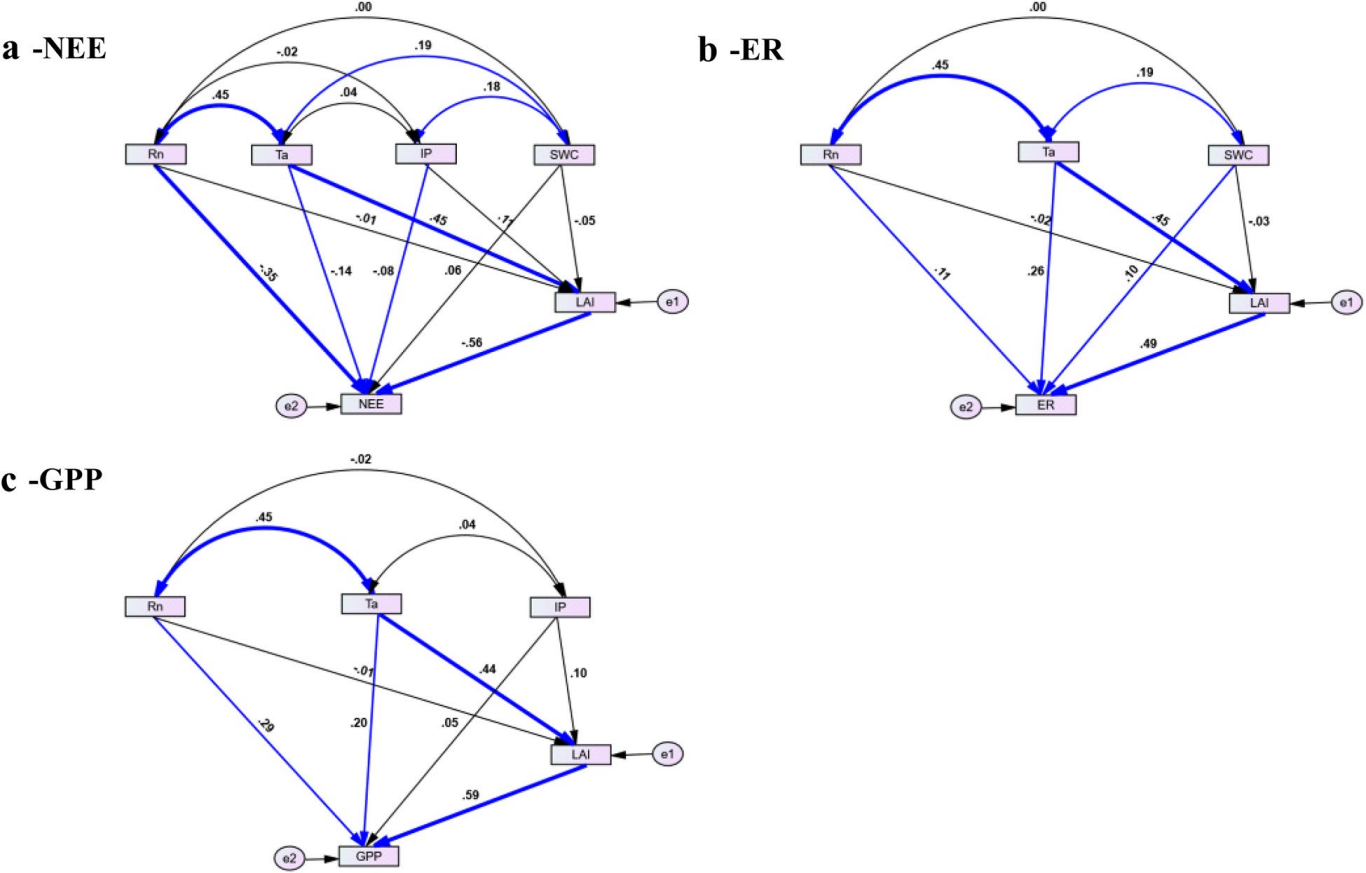
Structural equation models for daily net ecosystem echange (NEE). ecosystem respiration (ER) and gross primary production (GPP) during the growing season in 2014–2018. (Values on arrows are the standardized path coefficients (SC), representing standardized total effects of climatic factors on carbon and water vapor fluxes. The line weight represents the standard effect sizes, and the black line represents the relationship is not significant, The thick blue line represents a very significant correlation, p < 0.01;The thin blue line represents a significant correlation, p < 0.05)

Many studies showed that radiation, air temperature, precipitation, soil moisture content and LAI are the main factors affecting carbon flux during the growing season in different ecosystems [2]. Our study showed that LAI had significant effects on NEE, ER and GPP, which indicated that the growth status of crops plays a crucial role in influencing carbon flux during the growth period (Fig. 8). Through path analysis, the study showed that LAI is the leading factor (59%) affecting the change of NEE in the maize growing season, followed by net radiation. In addition to LAI, air temperature was also a major control factor that drives the seasonal change of ER. GPP is mainly affected by LAI and net radiation. This is consistent with many published studies [16, 27, 42]. LAI is an indicator closely related to the growth process of crops and directly determines the intensity of photosynthesis and autotrophic respiration of crops. Environmental factors affect seasonal variations in carbon fluxes by influencing crop processes and providing available energy. A field experiment with winter wheat showed that LAI, air temperature, photosynthetic effective radiation and biomass weight accounted for approximately 80% of ER and GPP [41]. In another experimental study on farmland ecosystems, soil respiration in a winter field was found to be controlled by temperature, soil moisture and LAI [40]. This result is consistent with the previous finding of a positive impact of soil moisture on vegetation activity [27]. In a model-based study, temperature was identified as the major abiotic factor affecting soil carbon flux [17]. In addition to being influenced by temperature, soil respiration is controlled by LAI and soil moisture. Another factor that has strong impacts on carbon fluxes is management practices [8]. As tillage supplies substrates to the soil, the decomposition of soil microorganisms is enhanced, which leads to an increase in soil respiration.

### Annual carbon flux

The 5-year mean value of NEE in our study was − 579.09 g C m^−2^ (Table 4). We compiled data from published papers to compare NEE of maize among different areas (Table 7). We compared the NEE of maize among regions with different climate and management practices. Research in the arid region of northwest China, Yingke station observations showed the mean value of NEE in 2007–2008 was − 626 g C m^−2^, which was higher than the mean value of − 527.09 g C^−2^ during 2014–2018 in our research [43]. This is due to the fact that the observation time at yingke included fallow periods, whereas our study was conducted only during maize growing season. In Europe, NEE in the Netherlands and Italy was − 597 g C m^−2^, respectively, while the value of NEE was − 186 g C m^−2^ in France, in Italy the NEE was − 473 g C m^−2^. Due to the different growing environment of maize, there are great differences between maize field NEE. In order to analyze the impact of farmland management measures on carbon fluxes, some researchers have conducted analyses. In Nerbraska, USA, the results of the study showed that the NEE value of irrigated maize was lower than that of rainfed maize [39]. The results of the study on mulching and non-mulching showed that the NEE of maize field after mulching was smaller, indicating that the mulching could absorb more CO_2_ from the atmosphere [16].

**Table 7:** Comparisons of annual net ecosystem productivity (NEP), gross primary productivity (GPP), ecosystem respiration (ER) and ER/GPP for maize ecosystem in different regions

No.	Site	Latitude & longitude	Vegetation	Temperature	Precipitation	Period	NEP	GPP	ER	ER/GPP	Sources
				(℃)	(mm)		(g C m^−2^)	(g C m^−2^)	(g C m^−2^)	(%)	
1	Wuwei, China	37°52′ N, 102°50′ E	Spring maize	7.8	118	2014	630	1115	485	44	This study
					112	2015	729	1705	975	57	
					82	2016	569	1422	853	60	
					98	2017	373	1069	694	65	
					156	2018	527	1252	725	58	
2	Shouyang, China	37°45′ N, 113°12′ E	Spring maize (mulching)			2013–2014	579	1459	880	60	[16]
			Spring maize (non− mulching)			2013–2014	518	1370	852	62	
3	Nerbraska, USA	41°09′ N, 96°28′ W	Irrigated− maize			2001–2008	767	1796	1029	57	[39]
			Rain− fed maize				664	1536	872	57	
4	Wageningen, Netherlands	51°59′ N, 5°38′ E	Maize	10.5	803	2007–2008	332	1982	1652	83	[21]
5	Lamasquere, France	43°49′ N, 01°23′ E	Spring maize	12.95	620	2006	186	1286	1100	85	[8]
6	Northeast, Italy	46°00′ N, 13°01′ E	Maize			2007	473	1471	805	55	[1]
7	Yingke, China	38°51′ N, 100°25′ E	Spring maize	7.4	68.1	2007–2008	626	1567	941	60	[43]
8	Weishan, China	36°39′ N, 116°03′ E	Summer maize	13.3	532	2007	201	872	672	77	[26]
						2008	244	880	636	72	
9	luancheng, China	37°53′ N, 114°41′ E	Summer maize	12.5	480		143	556	413	74	[44]

The 5-year average ER/GPP ratio was 57% (Table 4), which is similar to the value of 60% reported in the Heihe River basin [43]. Research in the Wageningen, Netherlands, and Lamasquere, France, showed that the ER/GPP ratios in these regions were higher than those in other regions, with both sites having ratios of more than 80% [8, 21]. Suyker et al. (2012) showed that despite differences in NEE, GPP and ER between irrigated maize and rain-fed maize, the ER/GPP ratio of both types of maize was 57% [39]. This value is very close to our results. Another study showed that the ratio of ER to GPP in mulching spring maize was lower than that in non-mulching spring maize; although film mulching increased ER, GPP also increased [16]. Studies of summer maize in northern China have found that the ratio of ER to GPP was greater in summer maize than in spring maize, exceeding 70% [26, 44].

Farmland is different from forest, when the crops are ripe, crops have to be harvested. Therefore, when determining whether the farmland is a carbon source or a carbon sink, we need to consider the carbon emission exported by harvesting crops. During the growing seasons, 579 g C m^−2^ (Table 3) was sequestered by uptake of CO_2_ from atmosphere. The amount of carbon exported from field was 813 g C m^−2^ (Table 2). this implies a carbon loss of 234 g C m^−2^ from soil during the growing season. It should be noted that the amount of carbon input to the soil from the harvested residues has been taken into account in the calculation of the carbon output when ploughed into the soil. Compared with other regions, we found that all the other sites except shouyang were represented as carbon sources (NBP is positive) to varying degrees. This was because in shouyang, straw was used for returning to the field, leaving the rest of the field except the seeds [15].

The above results show that both growing environment and farmland management measures had significant impacts on carbon fluxes. More than half of the carbon dioxide fixed by crops through photosynthesis was returned to the atmosphere through ecosystem respiration in maize ecosystem. In agriculture, straw mulching can effectively reduce carbon loss.

### The uncertainty analysis of carbon flux

In the process of carbon flux observation and simulation, due to the complexity of the underlying surface and the limitation of meteorological conditions, the research results still have great uncertainty. There are three main sources of uncertainty in this study: (1) Observational uncertainty. Eddy covariance systems to observe the carbon exchange of terrestrial ecosystems require that the underlying surface is uniform and flat, but the actual situation often has some fluctuations and inhomogeneity. The installation of the instrument can not guarantee the absolute level, there is human error. The hyperstable state of the atmospheric condition in night may also affect the accuracy of the observations. All of these causes can lead to uncertainty in the observed results; (2) Model structual uncertainty. In the separation of net ecosystem exchange, we built a model based on the relationship between nighttime respiration and temperature, and then deduce the daytime respiration, without considering the influence of environmental factors such as radiation on daytime respiration, which has certain limitations; (3) Parametic uncertainty. The coefficients used in the calculation of carbon content in different organs of maize were obtained from the references, which has a certain degree of deviation from the actual situation, which will lead to the uncertainty of calculation.

## Conclusion

We measured the carbon flux annually from 2014 to 2018 using an eddy covariance system. This 5-year study showed that carbon flux exhibited single-peak seasonal patterns during the growing seasons. The ratio of the ER to the GPP ranged from 44 to 62% in growing seasons of the 5 years, indicating that over half of the carbon fixed via photosynthesis was released back to the atmosphere by respiration. The seasonal vaeiation of GPP significantly affected the variation of NEE and ER in the growing season. Leaf area index was the most significant factor to control the seasonal variation of carbon flux in the growing season, followed by Rn and Ta. In addition, soil water content has a significant effect on GPP. The 5-year mean values of NEE, ER and GPP in our study were − 527, 734 and 1313 g C m^−2^. Taking into account C_e_, the annual NBP was − 234 g C m^−2^. These results confirmed that the use of straw to raise livestocks in the arid areas of northwest China had increased carbon emissions, leading to an increase in carbon dioxide emissions in the region. As the carbon balance of farmland varies greatly, it is highly sensitive to management measures such as tillage, mulching, fertilization and straw mulching. Future research should focus on the carbon fluxes of different farmland systems and their responses to management measures and climate change.

## Data Availability

The observed meteorological data are shown in Fig. 2, crop growth information is shown in Fig. 3, and carbon flux data is shown in Fig. 4.
